# Shared care and gender identity support in Primary Care: The perspectives and experiences of parents/carers of young trans people

**DOI:** 10.1177/13634593221138616

**Published:** 2022-11-26

**Authors:** Zowie Davy, Jack Benson, Abby Barras

**Affiliations:** De Montfort University, UK; York St John University, UK; Mermaids, UK

**Keywords:** healthcare assemblages, limit situations, primary care, shared care, trans youth

## Abstract

This article addresses the complex issues surrounding trans youths’ shared care perceived by parents in primary care settings in the UK. The analyses in this article draws on qualitative data derived from an online survey of 153 parents with trans children. Through the conceptual framework of healthcare assemblages, findings suggest that quality shared care for trans youth is based upon transient service relationships inherent in their healthcare—primary care, gender identity services, endocrinologists, and Adolescent Mental Health Services (CAHMS)—and, as such, this complexity must be understood better by GPs in order for quality shared care to be administered. We explored various blockages to quality shared care within primary care surgeries that produced limit situations, such as lack of knowledge, training, or experience with trans healthcare. One other key factor was that there were strong external forces that were limiting trans youths’ quality shared care in the form of abject depictions from beyond the consultation, which all produced negative effects. Despite these blockages, we also demonstrate how and where quality shared care is received. For instance, we show that continuity of care or treatment after an initial diagnosis or assessment contributes to quality shared care as too does personalized care to those youths receiving it. Overall, this research provides insights into the complex perceptions of parents about what quality shared care is and ought to be for trans youth.

## Introduction

Quality primary care work is shaped by the bureaucratic management of healthcare in complex organizational assemblages consisting of multiple players that co-produce (healthy and ill) bodies. Appropriate, competent and quality primary care services are understudied (Edmiston et al., 2016) and to our knowledge in the UK particularly scarce within trans and gender diverse children’s healthcare assemblages. For this article trans people are defined as: A trans male or trans boy is a person who was assigned a female sex at birth and is a male/boy. A trans female or trans girl is someone who was assigned a male sex at birth and is a female/girl. Many trans people may not feel to be either males/boys or females/girls and are somewhere within a spectrum of masculine/feminine sex/gender, or feel that they are no sex/gender, and this may vary over time and are commonly known as non-binary, gender non-conforming, gender fluid among others (Quinn et al., 2017), all of whom will be referred to as trans hereunder.

Despite personalized, routine and specialist gender related primary and secondary healthcare needs for trans children being reported to produce varying quality experiences (Clark et al., 2018), the quality of more personalized, routine and gender related primary healthcare needs and shared care requires more understanding. Moreover, because of the transient service relationships inherent in caring for trans people—primary care, gender identity services, endocrinologists, surgeons, and Adolescent Mental Health Services (CAHMS)—quality healthcare must be understood, we suggest, through the conceptual framework of healthcare assemblages of shared care, where continuity of care or treatment after an initial diagnosis or assessment that is truly patient-centered must be established alongside acknowledging the cultural, systemic limits and powerful forces circulating in each healthcare service event (Mur-Veeman et al., 2001).

We use the term healthcare assemblage, following Duff (2014), to describe that the social forces produce events that come together in primary healthcare assemblages and produce the ways that heterogeneous elements are organized in the formation of healthcare desires within societies. Healthcare assemblages shift and change through time and through space because of the ever-changing relationship between systems, doctors, and patients (Davy, 2021). This acknowledgment of healthcare assemblages in healthcare systems permits more refined analyses of the relations, affects, and events for shared healthcare for trans people and can show how they are produced.

The healthcare system is also important to contextualize in any research to garner a better understanding of quality shared care for trans people. Quality healthcare is a dynamic phenomenon often in a state of flux precisely because of human and non-human interaction through time and in particular healthcare spaces and systems (Bell et al., 2002; Higgins, 2002). There is to date little understanding about the opportunities and barriers to quality shared care in the UK for trans children and their advocates when they present to GPs. Producing deeper knowledge about these healthcare assemblages is a pressing issue because there has been an increase in trans youth presenting to all healthcare services. While we do not know the numbers presenting to primary care, we can garner a conservative figure from the number of referrals to gender identity clinics. There were 1981 in 2016/17, 2563 in 2017/18, 2743 in 2018/19, 2748 in 2019/20, and 2383 in 2020/21 (Gender Identity Development Service, 2021). A recently disseminated freedom of information response from the Gender Identity Development Service reported that the current number of patients awaiting their first appointment at the clinic is 7902 patients with 5256 patients being accepted since December 2019.^1^ The children and youth population in the UK is approximately 15,620,000 and thus a conservative estimate of trans youth accessing primary healthcare is approximately 1 in every 2000 children that will potentially require some combination of shared care services.

This paper draws on empirical qualitative data from adults who advocate for youths who are trans. We describe the experiences of parents/carers and their understanding about quality shared care that is and is not produced in primary care. In this paper, we particularly explore quality, patient-centered shared care and the desires of the parent/carer population that manifest within primary care surgeries in England, Scotland, and Wales. We do not intend to aggregate how well or how bad primary care develops shared care with their patients, but to look at how various assemblages produce, or not as the case may be, quality shared care for trans youth.

## Quality in primary healthcare for trans people

Over recent years according to NHS England (n.d.), more convenient access to care and an increased number of GPs and practice staff are operating more successfully with hospital specialists and mental health care providers.^2^ As with quality primary care for other patients, trans care has been studied in a range of areas, although much more is required (Edmiston et al., 2016; Goldstein et al., 2019; Greene et al., 2017). There is little research on quality primary trans healthcare. What there is, is often placed within a broader aggregation of LGBT community health improvement models, which tends to minimize the heterogeneity of trans people, their health issues and diverse care desires.

In the UK, a GP is usually the first medical professional sought for those experiencing “gender issues” and/or desire to start the process of medical transition. Although there has been a greater recognition by the medical community of the gender health needs of young trans people, this has rightly drawn attention to several less than satisfactory approaches toward this population (Crowley et al., 2021; Pearce, 2018; Vincent, 2018). There is emerging evidence showing improved outcomes and psychosocial functioning when trans youth commence their healthcare within gender clinics (Guss et al., 2019); however, according to James et al. (2016) in the United States and Davy (2011), in the United Kingdom, primary care consultations are fraught with concerns from trans people and their parents/carers about discrimination, maltreatment, and not being taken seriously together with GPs having little experience or none at all about the processes of referrals or the medical and treatment protocols, bridging medications, potential risk indicators, or how shared care can be developed with a range of healthcare providers (see Teti et al., 2021).

There are a few notable primary care studies that have suggested that GPs must understand that they will likely encounter trans people at some point in their practice who may need specialist gender related care and routine healthcare uncoupled from it, whether it is obvious that they have met or treated a trans person before and of course not being transphobic (Feldman and Goldberg, 2006; Shires et al., 2018; Willoughby et al., 2010). Others have suggested that continuous quality improvements in trans healthcare (Ding et al., 2020), simulation of medical student consultations with trans actors rating the communication, sensitivity, and depth of questioning and how feedback is reflected on by learners (Bockting et al., 2004; Greene et al., 2017), the support for courteous and friendly staff engagement with trans patients (Johnson et al., 2020), electronic consultations (Potapov et al., 2021), and improvements in cancer screening (Kiran et al., 2019) are all required to improve quality shared care. From this research, primary care for trans people must be individualized, but also informed by the knowledge of potential risks, behaviors, and needs in a heterogenous group of patients (Davy and Siriwardena, 2012; McManara and Ng, 2016). Despite these findings, patient satisfaction surrounding shared care is a particularly difficult area to measure and manage due to varying baseline levels of expectations. This complexity suggests that we must understand quality shared healthcare through the lens of itinerant factors that produce healthcare events that are conducive or not to its quality.

## Shared care

According to the UK’s General Medical Council, contemporary shared care is conceptualized as decisions being made about the responsibility for

continuing care or treatment after an initial diagnosis or assessment. This responsibility should be based on the patient’s best interests, rather than on convenience or the cost of the medicine and associated monitoring or follow-up. Shared care requires the agreement of all parties, including the patient. It’s essential that all parties communicate effectively and work together (General Medical Council, 2021: n.p.).

In the UK, trans people’s treatments are commissioned through specialized commissioning, however, there seems to be some commissioning issues impacting shared care. Barrett (2016), a clinician at a gender identity clinic in London, UK has accused GPs of faltering on what he calls joint care, avoiding the accepted term “shared care.” A shared care model is used for specialist treatments delivered and monitored in general practice, with responsibility shared with specialists. However, this is resourced by clinical commissioning groups rather than the special commissioning funds available for trans healthcare. Luke (2016) in response to Barrett’s piece argues that if NHS England and gender identity clinics wish to work with GPs they need to commission the full package of care from the specialized commissioning pot. Luke (2016) accuses the gender identity clinics of planning to leave health interventions at the behest of GPs, including the commissioning costs of endocrine interventions and accompanying tests that are recommended (World Professional Association for Transgender Health, 2012).

## Endocrinology and other shared care interventions

In the UK, trans children under 18 years old must generally be referred to the Gender Identity Development Service (GIDS) and be seen by a multidisciplinary team in order to be assessed for gender dysphoria before they can be referred to endocrinological services, if so desired (NHS England, 2020). For those trans youth who are at the stage at which they wish to suppress their puberty with gonadotrophin releasing hormone analogs (GnRHa), detailed assessments by the multidisciplinary team, usually over three to six appointments, over several months, may then result in being referred to a consultant endocrinologist to see if they can take puberty suppressants (NHS England, 2020). This usually takes place as they reach Tanner Stage 2–3 puberty. Ongoing psychological support is also provided. From the age of 16, youths who have been on puberty suppressants for 12 months may be given sex/gender-affirming hormones. For those youths who access GIDS from the age of 16 will be similarly assessed and may be referred to see if they can take sex/gender affirming hormones. If the child reaches 17 years old during the assessment period, they may then be seen in an adult gender identity clinic or will be referred to one from GIDS.

Once the care pathway is established primary care should take over the day-to-day management of endocrinological commissioning. No matter where endocrine treatments and tests take place or from which commissioning pot it is derived, the World Professional Association for Transgender Health (2012: 43), which NHS England uses as their benchmark for the baseline standards of care, suggests that the primary care provider should work with specialists to assess the patient’s current hormone regimen for their safety levels, contraindications and if necessary refer back to the endocrinologist to replace with safer medications or doses if necessary. However, there is some contradictory advice from the General Medical Council (the regulatory body for doctors in the UK) regarding endocrinological interventions for gender dysphoria. Sustanon—testosterone is the only intervention that is licensed by the UK Medicines and Healthcare Regulatory Agency for gender dysphoria, whereas estrogens and GnRHas are not. Specifically, however, all these interventions are not prescribed to alleviate gender dysphoria, because firstly, not all trans people suffer from gender dysphoria (American Psychiatric Association, 2013; Davy and Toze, 2018), although a reduction in distress about bodily developments may be a correlative effect. Secondly, in the case of puberty suppressants, they are prescribed because the suspension of puberty provides youths the time needed to consider a complex situation, without the accompanying distress that is reportedly caused by the actual and imagined physical changes to the body (Davy, 2021), and for helping to produce a physical appearance that makes it possible for them to live better and healthier lives (Kreukels and Cohen-Kettenis, 2011).

Similarly, sex/gender-affirming hormones are an important part of the esthetics of sex/gender reassignment. Despite this, doctors can prescribe unlicensed medicine if they are “A. satisfied that there is sufficient evidence or experience of using the medicine to demonstrate its safety and efficacy. B. take responsibility for prescribing the medicine and for overseeing the patient’s care, monitoring and any follow up treatment, or make sure that arrangements are in place for another suitable doctor to do so. C. make a clear, accurate and legible record of all medicines prescribed and, where you are not following common practice, your reasons for prescribing an unlicensed medicine” (General Medical Council, 2022b: n.p.), and further state that “it would not be acceptable to refuse or delay treatment because you believe that a patient’s actions or lifestyle have contributed to their condition” (General Medical Council, 2022a: n.p.).

These affirmative approaches are not being fully met in GP practices in the UK as one in five GPs refuse to prescribe hormones for trans people despite obtaining guidance from GICs, suggesting that they are too afraid, that they do not have the experience or confidence, or feel that the treatment is too specialized (Barrett, 2016), and as we will see below, sometimes defer support at the surgery. To our knowledge there is no other published research that addresses trans youth’s shared care issues in the UK. We turn now to the current study to look at the reported issues playing out in shared care assemblages in the UK.

## Methodology

In this article, the following research questions will be addressed: (1) What are the shared care experiences of parents/carers advocating for their trans children? (2) How do patients perceive GP’s knowledge of gender identity and relevant medical pathways? (3) How do patients perceive GP’s attitude toward transgender patients? (4) What are the key areas for improvement within primary care in relation to shared care?

### Survey designs

Building on previous qualitative research with parents of trans children (Davy and Cordoba, 2019), we assumed that a survey would be useful for reaching a wider population, but not adequately inductive, and thus complex enough to enable us to produce a reflexive, recursive, and rhizomic account of participants’ healthcare events in GPs’ surgeries. Nor would we be able to offer experiential data that draws out the agentic nature of healthcare events, which we wanted to explore following Deleuzian inspired healthcare theorists (Duff, 2014; Fox, 2011, 2016; Fox and Alldred, 2018) who demonstrate that human and non-human forms of power are produced, but are ongoing, illustrating how the economic, epistemic, and biologic discourses within health systems are always in processes of becoming. That is, they are never static. Bodies, health bodies, monitoring bodies, educational bodies, and so on are dynamically produced through the colliding of different healthcare assemblages (on an ongoing basis). As such, health professionals and patients must continuously manage multiple forces within healthcare, such as science, economics, education and, ever more so now, patients’ desires (Davy, 2021).

As such, for this research the surveys were designed with the aim of collecting a mixture of quantitative and qualitative data. We incorporated free text boxes to the survey so that participants could provide more detailed contextual information about their experiences with GPs and offer a range of responses so that we could identify desires that facilitate the production of quality care and any recommendations for service improvements. We developed two similar surveys to produce youth and parents/carers (separately) experiences of primary healthcare and attitudes toward their GPs. The parent and carer survey consisted of 52 items with 27 open text boxes and the young person survey had 53 items with 26 open text boxes. They consisted of:

1. A demographic section about their age, sex assigned at birth, gender identity, country of residence (England, Scotland, Wales, and Northern Ireland), ethnicity, religion/spiritual beliefs, where they lived and where their general practitioner in located (village, small town, large town, city), the length of time registered at the primary care surgery and whether it was an NHS or private practice.
2. A range of questions about trans healthcare provision, perceived knowledgeability about trans healthcare from physicians and staff and experiences of being trans within the primary care settings and their integrated (shared) healthcare desires and needs.

The online surveys were conducted from April 2020 to August 2020.

### Sampling

Parents/carers of youth were primarily recruited through Mermaids, a charity that supports families with trans children exploring their gender identities. Access was agreed with the CEO of Mermaids, with approval granted for information about the study to be publicized as many times as necessary via Mermaids’ online forums and other social media platforms. The online presence of Mermaids allowed us to share the research information detailing the call for participants in multiple formats to ensure wider accessibility, including written materials, posters, and a video. A supplementary snowballing technique was employed. Participants who were given the link to the online survey could, if they wished, send the link to other parents/carers of a young trans people they knew who may have been interested in contributing to the study.

### Analytical framework

The qualitative survey data that we report on in this article was analyzed using a form of thematic analysis (TA) in NVivo.^3^ We call this TA macro-thematic assemblages. Following Braun and Clarke (2019), our approach to TA was to reflect the data as creative, reflexive, and subjective, with the researcher’s subjectivity understood as a resource for producing meaning while viewing these as territorializing, context-bound, temporarily aggregated and non-static. We acknowledge that the research machine that we produced (survey questionnaire, sampling strategy, and our own affectation on the data) will inevitably reveal quasi-aggregating affects in this article that may simplify the events and assemblage affectations from the participants, but suggest that this is a necessary feature in our social inquiry for it to be understood (see Fox and Alldred, 2014). As such, we understand that our research-assemblage interacts with the micropolitics of the healthcare events in non-predictive ways and that our “aggregating and territorialising research-assemblage will dominate the affective flow in [. . .] remaking the event-assemblage [. . . and] aspects of the event’s affective flow will remain within [the] research output” (Fox and Alldred, 2014: 411). The intention was to understand the singularities within broader macro thematic assemblages produced by parents/carers about their child’s healthcare in relation to trans related shared care. The extracts presented below, and our analysis however seeks to remain responsive to the material, affective human, and non-human forces that shape and affect the stories that people told us.

### Ethics

Every consideration was made to ensure that all the participants were fully informed about the online research, by providing complete and transparent information. Additional care was taken to ensure that any other support that they may need was signposted to them at the beginning and at the end of the surveys. We asked all participants to take some time to decide whether they would like to participate and encouraged them to discuss this decision with a trusted person where possible. The survey undertaken was anonymous. All participants received a debriefing sheet at the end of the survey, and the contact details of the researchers and the team at Mermaids for all enquiries about the research and dissemination. General ethical considerations were also adhered to, such as the anonymizing of the free text responses in the survey, the maintenance of confidentiality and data storage security. Ethical clearance (HLS FREC Ref: 3569) was granted 5th May 2020 by the Health and Life Sciences, Faculty Research Ethics Committee, De Montfort University, Leicester.

## Results

One hundred fifty three parents/carers responded to the survey. There was 1 participant in the age range of 18–24, 2 between 25 and 34, 30 between 35 and 44, 90 between 45 and 54, 19 between 54 and 64, and 1 between 65 and 74 years of age. Ten declined to answer and 10 skipped the question. There were 12 male, 128 female, 1 non-binary, and 1 other and 1 declined to answer and 10 skipped the question of which 140 were cisgender, 2 were not, 1 declined to respond, and 10 skipped the question. One hundred twenty-seven participants lived in England, 5 in Wales, and 11 in Scotland and 10 skipped the question. Twenty-eight participants lived in a village, 54 in a small town, 23 in a large town, and 38 in a city and 10 skipped the question. There were 6 participants of mixed heritage, 116 white British, 5 white Irish, 16 white other, and 11 skipped the question of which 39 were Christian, 3 Jewish, 10 agnostics, 86 had no religion, and 10 suggested that they were pagan, spiritual, or humanist. Ten skipped the question. We did not ask about educational attainment or profession. For this article, we report on the free text data provided by parents/carers about quality shared care. We have decided to focus on shared care relationships with quality healthcare, because the responses about quality shared care was relatively widespread within the patient-physician-system assemblage, despite some parents/carers suggesting that they did not require shared care at the time of the research.

## Getting it right

Twenty-eight participants suggested that primary care was not an issue for them and were generally happy with the patient orientated (shared) care that they were receiving from their GPs and other clinicians. One parent said:

My son has had some complex health issues alongside his transition and other than a few hospital admissions his GP has seen him every two weeks for four years. He has shown empathy and understanding, has worked collaboratively with other health professionals and most of all has individualised his care knowing what is important to him and what will help him.

Highlighted by this parent/carer is the importance of an empathic relationship that focuses on the child’s wellbeing and life affirmation while working with other health professionals in the provision of care. This affective assemblage seems to produce positive effective care that this parent/carer is happy with, not least because it is important to the child. This “individualized” approach toward the child is important for understanding him in his singularity and the transitory nature of his life. Some research (Voss and Simons, 2021) from the US also suggests that clinicians need to individualize trans youth so that multiple aspects of their lives can be considered in relation to their care as this will enable better outcomes. This enables everyone involved to focus on prospective possibilities and actions. Making the consultation event more personalized moves away from therapeutic protocols that standardize treatment trajectories, which is ubiquitous in trans medicine.

Similarly, healthcare assemblages, consisting of human and non-human forces through time in relation to blood monitoring and utilizing the skills of other doctors to help support safe and appropriate levels of exogenous hormones or puberty blockers, contribute to quality shared care health events. The GPs mentioned in the extracts below are understood to be contributing to quality shared care by desiring the co-production of events while liberating elements that can enter new patient-GP-system relations.

[GP] took us away from the private provider, referred my daughter to an endocrinologist who determined an appropriate prescription and then took over her prescriptions and monitoring. They have taken the necessary steps to ensure my daughter is properly cared for in relation to her gender care. It has given her quality of life that she otherwise would not have had.

My child presents as Non-binary, I don’t know if the GP really understands this, but has never questioned the use of gender-neutral pronouns and has consistently shared care and been supportive.

The GP was respectful of everything my son shared, signed the referral to adult services without question and agreed to shared care with GenderGP^4^ later on with no difficulties. It may have helped because I sent him lots of information ahead of our appointments with him telling him what we would be seeing him about and providing background information to the services we required access to.

The co-development of these moments of quality shared care acknowledges times, spaces, and desires for these parents/carers, rather than shutting them down through a moralizing consciousness as we will see below. Deleuze (1988) suggests that while things can be objectively good or bad, they are not contingent upon a moralizing consciousness. They are good and bad because human and non-human bodies act upon another and produce good or bad affectations that either increases or decreases the power to act, or as Deleuze and Guattari (2004) would put it, it is good when the affects open a line of flight that was previously blocked and join with it in composing a more powerful body.

A few participants suggested that despite the lack of training or experience with trans healthcare, their GP overcame this, for example:

I have heard lots of stories about trans young people and their families being unsupported by their GP. Ours, though they often don’t know what to do, are willing to find out, have made referrals, have accepted shared care with NHS endocrinology, organized changing patient records and been lovely and supportive in person to my son who doesn’t like receiving injections.

The GP was reported to have identified means for processing referrals and attending to more systemic factors, such as changing the sex/gender marker and name of the patient resulting in an affirmative, patient-centered environment. Gender affirmation according to many participants involve clinicians asking about their patients’ pronouns and consistently using them in all correspondence whereas when forms and other systems only include male/female options it erases other gender identities (Goldenberg et al., 2019). Acts that challenge the sex/gender scheme were understood as attempts at the rejection of state and institutionally imposed territorializations of bodies and within (internet-based) healthcare systems, and act as a critique of the over-coded system of sex/gender. These affirmations were also productive, enabling new ideas, ideals, and discourses to permeate the social space and social hierarchies at the surgery.

Nonetheless, shared care was also understood to be precarious depending on social, medical, legal, personal, and political assemblages that are produced momentarily through time. Forces assembling through time with different effects demonstrate the contingency of quality trans primary healthcare rather than being something that can be established in a series of set features. A list of set processes can achieve nothing of importance about how quality shared trans healthcare may be realized in life, and must be guided by “a relational achievement, as the effect of bodies acting together in force and sympathy” (Duff, 2014: 185). The following participants articulated with a sense of fortuitousness the precarity of forces and sympathy playing out in the surgeries that they visited:

The first GP was at our previous address before we moved house. They were supportive in theory when my daughter came out, but not willing to share care with a private specialist. We were already in the process of moving from Scotland to England so had to change GP a few months later and also get a new GIC referral in any case. We were lucky to find a very supportive GP in our new town.

I sent a ton of information ahead of the appointment including our almost completely filled out application form for GIC referral. This was because I had no idea if the GP knew anything about trans healthcare nor how supportive they would be (we were new to the surgery). I was pleased that they didn’t argue against anything my son said and later were very supportive about entering a shared care agreement. They clearly had no awareness of waiting lists however, asking my son three months later if he had been seen yet!

Having them do shared care with GenderGP but I do understand since they were struck off, it must be worrying for GPs. I also wish GPs were able to triage before Tavi[stock] as the wait is terrible, if my son could have had blockers 2 years ago that would’ve helped and maybe prevent the suicide attempt.

The first quotation above and several other participants suggest that the contingent affects that assemble in the consultation is like taking part in a lottery, which shifts trans primary care from minimum consistency of care as a standard of quality care (World Professional Association for Transgender Health, 2012), to one, as we will see below, based upon what reaction they receive and the form that a GPs’ recognition takes when encountering their patients. As with cisgender patients, to be taken seriously is a complex process (Frederiksen et al., 2009).

Taking a patient seriously acts as relational achievements for the patient and produces an understanding that their presentations through time are valid while respecting, listening, understanding, confirming, and accepting them. Each of the following extracts demonstrates this, not in terms of fixed knowable essences or identities but in terms of events.

Our GP has been understanding & supportive. The surgery has also agreed to support with the GenderGP care (e.g., blood tests etc.).

Our GP said he had supported many other trans individuals, had a good sensitive and proactive approach and was happy to share care with both NHS and private Gender Specialists including prescribing cross sex hormones when indicated.

The relationships mobilize or contain entities, bodies, and subjects in ways that render each as distinctive kinds of individuals in the moment of their expression in the event (Duff, 2014: 48). As Vincent (2018) suggests trans individuals may have different needs in terms of medical care and medical pathways and therefore flexibility to incorporate these needs indicated to the parents/carers quality shared cared was being administered.

Trans youth, their parents/carers and GPs working with gender specialists is clearly working for some, leading to treatment(s) negotiated with a view toward the patient’s and parent/carer’s healthcare desires.

Our GP has supported us and our child through the whole process and has been happy to offer shared care with The Tavistock & Portman GIDS.

Our GP respects my child and has never questioned his capacity or understanding. As long as we have had a consultant to oversee treatment and to make sure things like blood tests and bone density scans are done regularly our GP has happily shared care (NHS and Private) and respected my child’s choices without question and supporting gender affirmative care.

While these responses indicate events taking place for each patient through time in relation to shared care, such diverse assemblages produce micro-relational ways of understanding trans primary care service provision. The desire for shared care is manifest through the choices available to each person in the assemblage that takes time and a will to produce.

Perseverance, negotiation along with utilizing trans healthcare process knowledge all challenge the power of medical professionals in the distribution of healthcare rights (Davy et al., 2018; Hines et al., 2017). Various blockages however produce limit situations for quality shared care, it seems. These blockages were eventually surpassed according to the extracts above. Individualizing the understanding of quality shared care and despite the limit situations that parents/carers and their children are experiencing, seems to be establishing ways for trans and sex/gender expansive children to co-produce the events in some GP’s surgeries. These events do not disavow the existence of the child and parent/carers’ desires. Moreover, GPs are not subjecting themselves to *certain* repetitive commitments to sex/gender norms, which in turn supports the co-production of new medical pathways.

## Human and non-human limit-events for shared care

Despite some GPs’ commitment to support, some parents/carers said that abject depictions can be projected onto trans children from beyond the consultation, which infuse forces that affect each event.

Although my GP wasn’t dismissive of having joint care between a private clinic, she did not want to be drawn into this conversation and instead said this would be something the Mgrs. would need to think about and make decisions on. I wonder why in 2020 these decisions haven’t already been put in place.

Whilst our current GP has been great, two other GPs within the same practice have refused care or been resistant to prescribing. It would have been helpful if all GPs within the same practice we’re as knowledgeable, understanding, and supportive as our current GP - there are such hugely different attitudes and knowledge bases from GP’s even at the same practice.

Although they want very much to help, my GP cannot support us in the way they want to as we are still on the endless wait for the Tavistock, 26 months plus later. Their insurance does not cover them to continue prescribing hormones and blockers as advised by GGP since being suspended. And as they are the only under 18 provider we can use, we are stuck with private care. The waiting time is clearly unacceptable, the lack of alternatives woeful and the costs and stress of trying to deal with it all is appalling.

These participants’ extracts reflect several other participants in the study who all identify both human and non-human processes and macro-structural factors that affect quality shared care health outcomes. GPs and managers who are reluctant to support trans children exert their desire onto colleagues who wish to be supportive in the consultation event, with differing degrees of force. Similarly, factors outside of the surgery, such as clinic waiting times, structural protocols, and economic factors impact the perceived quality of shared care.

## Unwilling to share care with private providers

One other external set of events that has impacted the provision of quality shared care and which has been referred to multiple times in participants’ extracts is the private trans healthcare provider GenderGP. Helen Webberley was providing private care to some trans and sex/gender expansive children and prescribing GnRHas. She had her license to practice revoked. As a result of a widely publicized case, being removed from the NHS’ GP list, and because of an earlier conviction for not being registered to prescribe from her private practice, she was suspended until these matters were looked at by the Medical Practitioner’s Tribunal Service (MPTS). Her partner continued to provide support, however, despite the GenderGP surgery being unaffected by any outcome of the tribunal due to different qualified owners of the practice, there are many reported refusals by NHS GPs to work with them. The following extracts highlight how parents/carers perceive the refusals:

My daughter has private care from GenderGP and 2 GPs have now categorically refused shared care or any form of support. The previous GP said, “We just don’t like them.”

GenderGP is the only private gender service that will help under 18’s, our GP refused to share care.

The GPs’ views so far have been very closed minded about private care, and it may be all we have. The shutters come down with force if I even mention it. Shared care is clearly never going to be on the cards with this practice.

In the UK patients are entitled to free NHS care even if you choose to pay for additional private care. There must be a clear separation between your private treatment and your NHS treatment. While this is a vague clause and can be interpreted in several ways, it generally means that you cannot combine different parts of the same treatment between NHS and private care. The healthcare assemblages for these participants however becomes an either/or limit situation resulting in the refusal to work with the private provider. Other participants, suggest that there are other forces entering the healthcare assemblages, such as an evaluation of risk.

## GPs’ risk aversion

It is widely accepted that medicine is dominated by “risk-thinking” (Rose, 2005: 14) that produces a context of checks and balances. Risks according to a few participants were not related to therapeutic contraindications, but because the child may change their minds about their gender identities.

The GP has refused shared care because (in their words) “if she changes her mind, she will sue us, not the gender clinic and not the hospital.” They have said they are happy to assist when she is older, but not whilst she is under 18.

I think it’s possible my GP thinks we may sue if something goes wrong. I’ve read the shared care agreement and don’t understand why they are concerned.

The whole issue of shared care needs to be reviewed. GPs seem to feel like they are taking on something that could be detrimental to their career, and they need to be educated as to how detrimental it is to the young people and how it is actually their duty to care for everyone equally no matter what treatment they need. And they need to understand that treatment for a trans person is as important as treatment for something like diabetes – it’s life or death for that person, not just a fad or like cosmetic surgery.

Participants understood the risk evaluations of providing shared care by GPs to be about protecting their jobs and reputation rather than understanding that their medical work is about informing patients about the evidence of risks and benefits and any other key information that a patient may need to understand more fully about a procedure. Risk thinking by these GPs is seen as forces that refuse the patient the freedom to actively fashion their lives and, as such, produces normative effects. Normativity, according to Deleuze (1992: 256), is a set of forces that halts someone’s “power of acting” and excludes movement, creativity, developments, and new styles of life and organization.

## Desire for quality shared care

Many parents’ desires for quality shared care are captured by the following extracts:

Full understanding of the huge mental impact of gender dysphoria as well as knowledge & understanding of gender identity, healthcare & transition. The importance of full support especially in shared care. Feedback to reduce waiting times to and increased provision of gender support services. Also, to review protocols to offer more timely personalised treatment.

Moreover, shared care should not be

a complete lottery when trying to find a GP that is knowledgeable and supportive about transgender care for children and young people. I know with absolute certainty that we have been incredibly lucky to find a GP that is willing to support both affirmative care and shared care within both NHS and private trans care settings. Even within the same GP surgery opinion was divided. I believe all GPs should have transgender awareness training, and better understand treatment pathways, waiting lists, where to find support. We will be moving house soon, which will mean a change of GP. If my child was not already established in adult care services, I absolutely know it would be stressful and difficult to find another GP as supportive as the one we have now. [. . .] We have been lucky. Getting good affirmative treatment should not come down to luck or lottery.

Similar to research in Australia (Strauss et al., 2022), there was a sense that finding a GP who was knowledgeable about gender diversity, trans healthcare needs, and with experience working with trans people should not come down to luck. The desire for quality shared care for trans children according to the parents/carers above is contingent upon multiple factors, such as understanding what gender dysphoria may entail, how care pathways work and can be personalized to their children, compassionate physicians, parent advocacy, facilitating managers, and institutional mechanisms that work. In sum, an understanding about the individualized nature of healthcare assemblages that move through time and space, and through other contingencies.

## Concluding remarks

We have demonstrated how healthcare assemblages produce social forces within heterogenous healthcare events that organize healthcare desires for families with trans children. Moreover, we explored various blockages to quality shared care within primary care surgeries that produced limit situations, such as lack of knowledge, lack of training or experience with trans healthcare, and abject depictions of trans children from outside, which all produced negative effects. However, sometimes these blockages were eventually maneuvered through, seemingly establishing ways for parents/carers and their trans children to co-produce the events in some GP’s surgeries. Specifically, GPs, according to many parents/carers of trans children, should consider more appropriate and personalized responses to shared care, particularly because many are facing lengthy waiting times in the Gender Identity Clinics. Trans children’s and their parents’ desires for quality shared care must include access to a range of trans healthcare services from all GPs that are already being facilitated by many other GPs as a form of personalized shared healthcare, without, it seems, any issues. The sentiment from parents is there is a system that allows some and disallows others from quality shared healthcare that ought to be more equitably distributed in primary care.

## Notes

1. See https://www.whatdotheyknow.com/request/current_wait_times_for_tavistock#incoming-1746863

2. In the UK, the BMA has suggested that there were problems in primary care before the Covid-19 pandemic in that patient demand was increasing while the number of general practitioners was falling.

3. For results of the of the quantitative analysis, please see https://mermaidsuk.org.uk/wp-content/uploads/2022/04/The-Lottery-Of-Primary-Care-April-14-22-1.pdf

4. GenderGP is an online health and wellbeing clinic, which serves trans people of all ages and those who support them (see https://www.gendergp.com/).
